# Classifying intentions for carbon neutrality participation through latent class analysis

**DOI:** 10.1016/j.heliyon.2024.e40721

**Published:** 2024-11-28

**Authors:** Jae Eun Lee, Seol A. Kwon, Hyun Soo Park, Ke Zhang, Wei Lu, Lin Dong

**Affiliations:** a Department of Public Administration and Department of Crisisonomy, Chungbuk National University, Chungbuk, 28644, South Korea; b National Crisisonomy Institute, Chungbuk National University, Chungbuk, 28644, South Korea; c Department of Public Administration, Law School & Intellectual Property School, Sichuan University of Science & Engineering, Sichuan Province, 643000, China; d Department of Crisisonomy, Chungbuk National University, Chungbuk, 28644, South Korea; e Department of Public Administration, Chungbuk National University, Chungbuk, 28644, South Korea

**Keywords:** Climate crisis, Carbon neutrality, Climate crisis awareness, Public participation, Latent class analysis (LCA)

## Abstract

This study aims to classify citizens' intentions to participate in carbon neutrality activities, emphasizing the crucial role of public engagement in achieving sustainability goals amid the climate crisis. The climate crisis has rapidly become one of the most pressing challenges to global sustainable development, with carbon neutrality as a key objective. To understand variations in citizens' willingness to engage in carbon neutrality efforts, we conducted a Latent Class Analysis (LCA) on responses from 800 residents of Cheongju, South Korea, categorizing their willingness to participate in 13 different carbon neutrality activities. The analysis identified three distinct groups: 'Active Participation,' 'Limited Daily Life Participation,' and 'Passive Participation,' each reflecting different levels of awareness and practice in response to the climate crisis. The findings highlight that both individual awareness and peer behavior are significant factors influencing participation in carbon neutrality activities. The practical implications of this study involve creating targeted strategies to enhance public engagement in carbon neutrality, offering valuable insights for policymakers and climate advocates in developing effective interventions to promote a more sustainable society.

## 1 Introduction

Since the United Nations began addressing climate change through intergovernmental negotiations in 1990, global climate governance has seen three major milestones: the United Nations Framework Convention on Climate Change (UNFCCC) [1], the Kyoto Protocol [2], and the Paris Agreement [3]. The Kyoto Protocol imposed legally binding obligations on developed countries to reduce and limit greenhouse gas emissions [2], while the Paris Agreement allows each country to set its own carbon reduction targets based on its circumstances [3].

Despite these efforts, the World Meteorological Organization's 2023 Provisional State of the Global Climate report shows that by the end of October 2023, the global average temperature had risen 1.4 °C above pre-industrial levels [4]. The period from 2015 to 2023 has been the hottest nine years ever recorded, with many climate records being broken. This rapid warming is unprecedented, and extreme weather events are increasingly devastating lives and livelihoods around the world.

Climate change has emerged as one of the most critical challenges to global sustainable development, necessitating urgent and widespread action toward carbon neutrality [5]. Achieving this goal requires not only the implementation of technological solutions and policy measures but also significant changes in individual behaviors and societal norms. Citizens, as the primary consumers of life energy and the end-users of energy-intensive industrial products [6], possess substantial potential for energy conservation and emission reduction. Their individual lifestyles and consumption patterns impact multiple greenhouse gas-emitting sectors, such as the energy industry, transportation, and residential sectors [7].

To guide the transformation of citizens' lifestyles towards carbon-neutral behaviors, it is essential to understand the factors that influence their behaviors and how to increase their willingness to act. This study aims to classify citizens' intentions to participate in carbon neutrality activities, emphasizing the crucial role of public engagement in achieving sustainability goals amid the climate crisis. The climate crisis has rapidly become one of the most pressing challenges to global sustainable development, with carbon neutrality as a key objective.

Our research utilizes latent class analysis to categorize participants into three distinct groups based on their willingness to engage in carbon neutrality activities. This segmentation reflects different attitudes and readiness levels in the population toward adopting carbon-neutral practices. The study's insights into these dynamics are vital for developing targeted strategies to enhance public engagement in carbon neutrality.

## 2 Theoretical background

In recent years, digital technologies have emerged as a crucial driver for promoting sustainable development, focusing on minimizing energy waste and reducing carbon emissions [8]. Bibliometric analyses of carbon neutrality research indicate that technological innovations, including digital tools like AI, IoT, and big data, have significantly enhanced energy utilization efficiency, particularly in comprehensive experimental zones [9,10]. These technologies play a key role in reducing emissions in sectors like energy, transportation, and manufacturing, highlighting their importance in achieving carbon neutrality. However, the effectiveness of digital empowerment in this area depends on various contextual factors, such as geographical location, economic development levels, and energy structures, which shape the overall impact of digital tools on the carbon cycle [8,[11], [12], [13], [14]].

Furthermore, the potential benefits of disruptive digital technologies in areas such as carbon replacement, carbon reduction, and carbon capture, utilization and storage (CCUS) have been extensively explored in existing research [15]. Nevertheless, due to the inherent uncertainty of environmental risks and the economic feasibility of projects, transforming energy consumption patterns and shifting to sustainable energy sources remain pivotal steps for many countries to achieve their carbon neutrality goals [16]. This demonstrates that a purely technology-driven approach is insufficient for achieving carbon neutrality; policy guidance is equally crucial [17]. Carbon pricing represents a prominent climate policy that has significantly influenced the energy industry's transition from traditional fossil fuels to low-carbon and clean energy sources [18]. It is unfortunate that concerns regarding the fairness of carbon pricing have resulted in a general lack of public acceptance, particularly among vulnerable groups [19].

From the perspective of climate governance, the achievement of carbon neutrality necessitates a unified effort on the part of global consumers, businesses, and governments [20]. While transitioning to a low-carbon energy system is essential, the cumulative impact of individual low-carbon actions can generate substantial momentum toward carbon neutrality across sectors. As climate governance evolves towards a decentralized model, it is vital to consider individual-level actions as a central part of this transition [21].

Despite significant efforts in technology and policy to achieve carbon neutrality, reaching individual carbon neutrality remains a major challenge. Studies have highlighted that factor such as insufficient motivation for low-carbon consumption, along with personal preferences, cognition, and willingness, play critical roles in shaping consumption behaviors [22,23]. This indicates that, in order to achieve personal carbon neutrality, it is initially essential to alter our perspective and assume the role of active participants in the pursuit of carbon neutrality, recognizing that it is a collective responsibility and obligation [24]. It is therefore imperative to strengthen public awareness and enhance societal enthusiasm for emission reduction. Public education and outreach efforts are essential in guiding individuals toward adopting low-carbon lifestyles, which are pivotal for achieving carbon neutrality [25,26].

Citizen participation is increasingly recognized as a cornerstone in climate change mitigation efforts [27]. The willingness of individuals to engage in low-carbon behaviors is a critical determinant of the success of carbon-neutral policies [28]. Previous research has explored various factors influencing eco-friendly behavior, such as repeat purchase intentions among eco-city residents [29], risk perception, personal practices, social norms [30], and perceived costs of environmental behaviors [31].

At present, research into carbon neutrality has developed into an interdisciplinary field, drawing upon insights from a range of disciplines, including environmental science, economics, and policy studies. Increased global collaboration and a focus on practical applications are evident in recent research trends, reflecting a commitment to real-world carbon neutrality strategies. Notwithstanding these endeavors, there is a paucity of research that disaggregates the willingness to engage in carbon-neutral activities and compares the influencing factors across different demographic groups within the context of "carbon neutrality." This study aims to address this gap by exploring how public willingness varies across different segments of the population and identifying the factors that influence these differences.

## 3 Methodology

### 3.1 Data collection

**Fig. 1:**
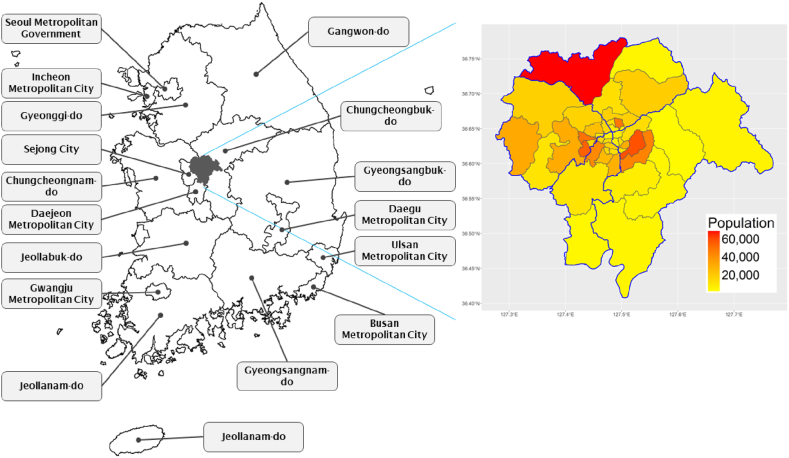
Cheongju-si administration map.

The research data was collected through an online survey conducted in October 2023, targeting residents of Cheongju city aged 18 and above. The survey explored various aspects, including perceptions of the climate crisis, awareness of climate crisis policies, understanding of climate crisis adaptation, general awareness of carbon neutrality initiatives, and perceptions of actions towards achieving carbon neutrality. A total of 800 respondents participated in this survey. Fig. 1 shows the Cheongju-si administration map.

### 3.2 Measurement of variables

#### 3.2.1 Willingness to participate in activities for carbon neutrality

Carbon neutrality is inseparable from public participation, whether the public can participate in carbon neutrality governance, what degree of participation and how to participate are important criteria for the construction of carbon neutrality diversified governance [32,33]. In response to the global climate crisis, public participation in carbon neutral activities is of more important practical significance to the process of climate change adaptation and mitigation, as climate warming is related to the environmental interests and economic well-being of the public [34,35]. Zhang et al. [36] pointed out that improving the knowledge level of climate change can motivate people to actively participate in carbon neutral education. When people consider whether to participate in carbon neutral activities, they are influenced by subjective normative factors, and they will fully weigh their own interests, gains, and losses when considering whether to implement carbon neutral activities [37], therefore, people's awareness of "carbon neutral" behavior and willingness to pay for carbon emission reduction are important factors that influence willingness to participate in “carbon neutral” behavior [38]. People's behavioral intention to participate in carbon neutrality is influenced by perceived behavioral control factors, the channels and modes of information dissemination about carbon neutrality will affect people's cognition of carbon neutrality [39]. Although concern and perception of climate change is steadily growing [40], but people do not always have a personal experience of the effects of climate change [41]. Some research pointed out that habitual inspiration has a greater impact on people's behavioral intention to participate in carbon neutrality [42]. At present, most people are influenced by traditional lifestyles, and their understanding of carbon neutrality and environmental protection is only formal, and there is not really form carbon neutral perception and behavior [43]. To achieve carbon neutrality, it is necessary to constantly update the energy system and industry system closely related to the public life, constantly expand the channels for the public to participate in carbon neutral governance and enhance the enthusiasm of the public to participate in carbon neutral activities [44,45].

In this study, the dependent variable - willingness to participate in carbon-neutral activities - was divided into four categories: energy, transportation, resource circulation, and others. Respondents' willingness to engage in carbon-neutral actions was measured within each category.

In the energy category, participants were asked about activities such as 'using high-efficiency energy products,' 'installing and using renewable energy systems,' 'efficient heating and cooling management (maintaining appropriate temperatures),' and 'improving home insulation.' For transportation, items included 'using public transportation,' 'cycling or walking,' and 'using or buying eco-friendly vehicles like electric or hydrogen cars.' In the resource circulation category, activities were 'avoiding single-use products,' 'reducing plastic use,' 'minimizing food waste,' and 'buying second-hand goods.' Finally, in the other category, items included 'choosing eco-friendly and low-carbon products, even if they cost more' and 'buying locally produced food.' Each of the 13 items was rated on a 5-point scale, from 'very difficult to participate' to 'actively participate.'

The dependent variable, "intention to participate in activities for carbon neutrality," was recoded into a two-point scale: "unlikely to participate" (encompassing "very unlikely to participate," "somewhat unlikely to participate," and "neutral") and "likely to participate" (including "likely to participate" and "very likely to participate"). The rationale behind this recoding is that utilizing a five-point scale would result in the formation of numerous latent classes, complicating the interpretation of the analysis results. Thus, to ensure model parsimony, a two-point scale was employed.

#### 3.2.2 Independent variables

(1) Awareness of the Climate Crisis

Climate crisis awareness is the degree of people's awareness of the crisis caused by climate change. Climate crisis has become a serious challenge to the sustainable development of global society [46]. The public has been aware of various environmental crises around us for years, but awareness of the climate crisis has only recently begun to grow [47]. But recent research suggests that public concern about climate change has declined [48], increased attention to climate crisis may help the public enhance prevention awareness and strengthen self-protection, thus effectively curbing the spread of climate crisis [49]. Therefore, in the face of the climate crisis, it is necessary to establish the idea and consciousness of normal crisis prevention and control and continue to improve people's awareness of the climate crisis is the focus of carbon neutrality.

(2) Practice of Responding to the Climate Crisis

Although countries around the world have different perceptions of the climate crisis, most countries agree that the climate crisis has brought serious impacts on human society and the natural environment and needs to take active practice to deal with it [50]. The public's identification and response to the crisis are also concentrated manifestations of the crisis awareness, only by strengthening the climate crisis practice that we can avoid paying a huge price in the climate crisis [51]. According to the “London Environment Strategy” point out that only 35 % of London's zero carbon target can be achieved by 2050 according to the existing emission reduction policies, while the remaining carbon reduction target can be achieved through the implementation of new policies and strengthening of existing policies, which shows the importance of practices in response to the climate crisis to achieve carbon neutrality [52].

For the independent variables, first, the perception of the climate crisis was measured. Respondents were asked, "To what extent do you think the climate crisis is affecting your daily life?" The responses were measured on a 5-point scale ranging from 'does not affect at all' to 'affects very much'. Additionally, regarding the current situation of the climate crisis, the question "Do you think we are currently in a climate crisis situation?" was asked, with responses measured on a 5-point scale from 'strongly disagree' to 'strongly agree'.

Regarding the practice of responding to the climate crisis, respondents were first asked, "How much do you think the people around you (family, friends, colleagues, etc.) are practicing actions for carbon neutrality?" Responses for this were measured on an 11-point scale ranging from 'not at all' (0 points) to 'always practicing' (10 points). Next, the question "Are you aware of individual-level actions to respond to the climate crisis?" was asked, with responses measured on a 5-point scale from 'not aware at all' to 'very well aware'. Additionally, for the question "Are you willing to bear costs and inconveniences to achieve carbon neutrality?" responses were measured on a 5-point scale, ranging from 'unwilling to bear costs and inconvenience' to 'willing to willingly bear costs and inconvenience for carbon neutrality'.

Control variables such as gender and age were also considered to examine the influence of each independent variable on the willingness to participate in activities for carbon neutrality, regardless of gender and age.

### 3.3 Research model

To classify the willingness to participate in activities for carbon neutrality, Latent Class Analysis (LCA) will be conducted. LCA is a model-based clustering method [53], first introduced by Lazarfeld in 1950 [54], as a statistical procedure for categorizing respondents into classes based on similar response patterns [55]. It involves determining the number of classes, calculating the probability of each respondent belonging to each class, and then assigning respondents to the class where they have the highest probability of belonging. LCA is commonly used to model preference heterogeneity in specified choice data, exploring the impact of individual heterogeneity on participation behaviour and willingness to participate [56]. Thus, this method is suitable for investigating research questions and hypotheses for classifying respondents based on a chosen variable [53].

**Fig. 2:**
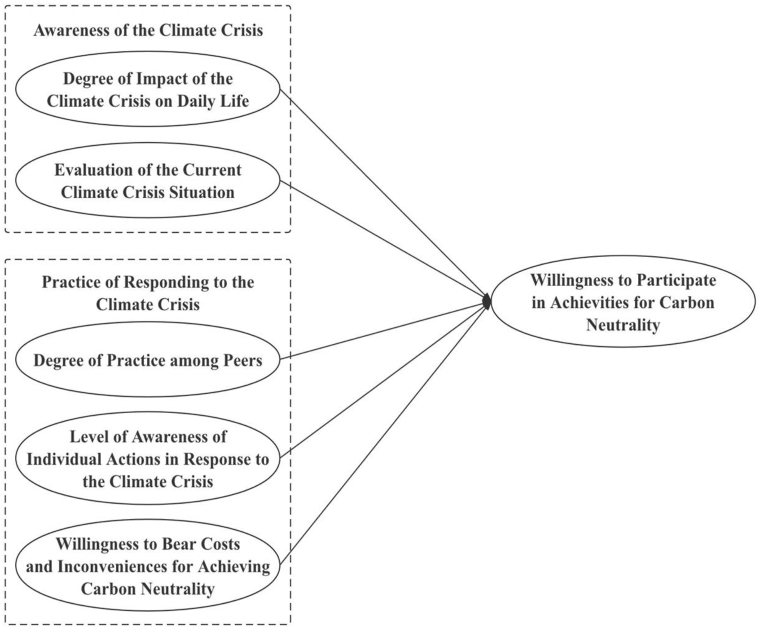
Research model.

The public's intention to participate in carbon neutral activities tends to differ to some degree due to differences in awareness of the climate crisis, and practice in responding to the climate crisis. The aim of this paper is to identify the factors that influence the willingness to participate in such activities and to explore ways to enhance public participation in activities for carbon neutrality. To achieve this, a public participation intention model for carbon neutral activities is constructed based on the LCA model, as shown in Fig. 2. Through latent class analysis, respondents can be categorized based on their willingness to participate in activities for carbon neutrality. This will allow for a comparison of the influence of climate crisis awareness and response practices across different classes. Such an analysis is expected to aid in developing strategies to enhance citizen participation in activities for carbon neutrality in the future.

#### 3.3.1 Hypotheses

Hypothesis 1 The degree impact of the climate crisis on daily life will affect the willingness to participate in activities for carbon neutrality.

Recent scientific studies have shown that the effects of climate change on food supply, health, living environment and more are changing our lives in unexpected ways [57,58]. Some study pointed out that residents in areas with severe climate disasters are more concerned about climate change, while residents in other areas with less impacts are less concerned about climate change [59]. It is assumed that the extent to which the climate crisis on daily life will affect people's willingness to participate in activities for carbon neutrality.

Hypothesis 2 The evaluation of the current climate crisis situation will affect the willingness to participate in activities for carbon neutrality.

More and more studies point out that evaluation of the crisis is an indispensable part of crisis management [60]. Constructing an assessment system for urban adaptation to climate change can quantify the city's level of coping with climate change and continuously enhance its ability to cope with climate change [61]. Climate assessment can effectively respond to climate change and reduce various meteorological disasters, especially it has an important impact on human activities [62]. It is therefore hypothesized that the evaluation of the current climate crisis will affect the willingness to participate in activities for carbon neutrality.

Hypothesis 3 The degree of practice among peers will affect their willingness to participate in activities for carbon neutrality.

Thomas et al. [63] pointed out that climate crisis also has different impacts in different countries, regions, and social groups. There are group differences in the public's cognition of low carbon. There are group differences in the public's perception of low carbon, and the living environment and the actions of people around them will affect the public's perception of low carbon [64]. The study between the degree of public concern and the epidemic of COVID-19 indicates that if the uncertain risk event occurs in a region far away from oneself, people lack the awareness to pay attention and understand in advance, and only when they realize that the risk event occurs in their own neighborhood will a lot of public attention be aroused [65,66]. It is therefore assumed that the degree of practice among peers will affect an individual's willingness to participate in carbon neutrality.

Hypothesis 4 The level of awareness of individual actions in response to the climate crisis will affect the willingness to participate in activities for carbon neutrality.

Widespread acceptance of the idea of carbon neutrality requires a long period of publicity and continuous low-carbon education [67]. The implementation results of various strategies to improve human risk prevention and crisis response capabilities will be changed due to differences in public perception [68,69]. Public awareness of climate change is cornerstone of climate crisis management. Chen and Li [70] pointed out that low-carbon awareness, low-carbon knowledge, and personal norms will have an impact on residents' low-carbon behavior. Therefore, it is hypothesized that the level of awareness of individual actions in response to the climate crisis affects the willingness to participate in carbon neutrality.

Hypothesis 5 The willingness to bear costs and inconveniences for achieving carbon neutrality will affect the willingness to participate in activities for carbon neutrality.

Levy and Patz [71] pointed out that the poor, especially those in rural and remote areas, will be more seriously affected by climate change. Yang et al. [72] pointed out that residents hold a positive attitude towards carbon trading policy, but when they are further asked whether they are willing to take green actions or pay to compensate for carbon emissions in daily life, the proportion of support drops [73]. Therefore, it is hypothesized that the willingness to bear costs and inconveniences for achieving carbon neutrality will affect the willingness to participate in activities for carbon neutrality.

## 4 Results

### 4.1 Descriptive statistics

The questionnaire received responses from a total of 800 participants. Among them, 298 were men, representing 37.3 % of the respondents, and 502 were women, representing 62.8 %. The age groups and response rates of respondents mainly have the following characteristics: 22 % were between 18 and 29, 31.8 % in their 30s, 26.5 % in their 40s, 15.4 % in their 50s, 3.4 % in their 60 and 64, and only 1 % of respondents were between ages of 65 and 69. Most respondents were between 18 and 59 years old, with the highest response rates from respondents in their 30s and 40s.

The survey questions mainly related to climate crisis awareness, practice of responding to the climate crisis and willingness to participate in activities for carbon neutrality. Table 1 shows the difference in the average willingness of each group of respondents to participate in different categories of carbon neutral activities. The group involved in the "Reducing Food Waste" (M = 4.20, *s* = 0.694) has the highest willingness to participate, followed by the group involved in "Efficient Operation of Heating and Cooling Systems"(M = 4.19, *s* = 0.775). The group that participates in "Use of High-Efficiency Energy Products"(M = 4.10, *s* = 0.766) has the same average value as the group that participates in "Reducing Plastic Use"(M = 4.10, *s* = 0.777). Additionally, the group that "Use and Purchase of Eco-Friendly Vehicles" has the lowest willingness to participate (M = 3.48, *s* = 1.061).

**Table 1:** Willingness to participate in activities for carbon neutrality.

item	Mean	Std. Deviation
Use of High-Efficiency Energy Products	4.101	0.766
Installation and Use of Renewable Energy Facilities	3.714	0.888
Efficient Operation of Heating and Cooling Systems	4.189	0.775
Enhancement of Home Insulation	3.878	0.877
Use of Public Transportation	3.813	1.022
Traveling by Bicycle, Walking	4.039	0.899
Use and Purchase of Eco-Friendly Vehicles	3.483	1.061
Avoiding Single-Use Products	4.046	0.794
Reducing Plastic Use	4.098	0.777
Reducing Food Waste	4.201	0.694
Using Second-hand Goods	3.871	0.884
Using Eco-Friendly and Low-Carbon Products	3.679	0.851
Using Locally Produced Food	3.896	0.785

### 4.2 Validation of the research model

As LCA is a model-based clustering method, to measure the relative fit of the model and to determine whether the LCA model describes the data in an accurate and useful way [74], we used the Bayesian Information Criterion (BIC) and Akaike Information Criterion (AIC) as model selection criteria. The BIC and AIC criteria are widely accepted as LCA model selection methods in previous studies [75] and it has been shown to be the most reliable criterion when deciding on the best latent class model [76]. Namely, lower values of AIC and BIC indicate a better fitting model [77].

**Fig. 3:**
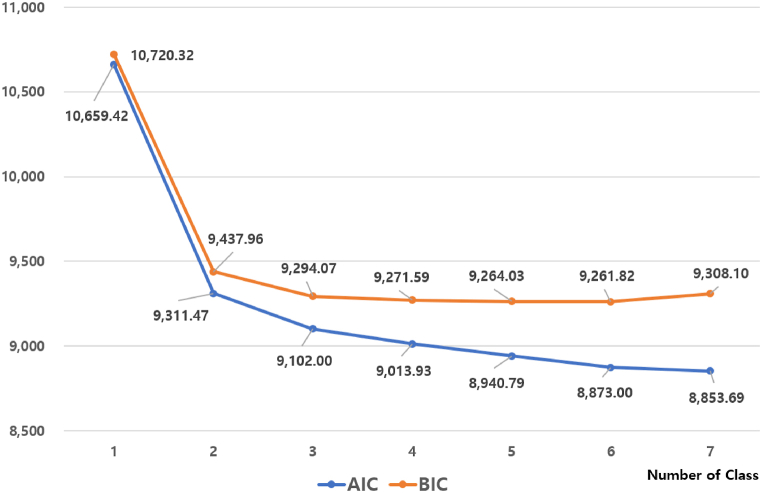
*Model-fit indexes for* LCA.

In this study, models were fitted for each of the seven potential categories of analysis in turn, using the dimensions of willingness to participate in carbon-neutral activities as indicators. In the data used for the analysis, there were no missing values in the indicator variables. Consequently, methods for handling missing data, such as deletion or multiple imputation, were not employed. Fig. 3 presents the fit indices for each of the seven class models. As the number of classes increased, the AIC and BIC decreased, indicating an improvement in model fit [78]. Of the seven class models, the latent class model consisting of 3 class was considered to be the best fit for the data. The 3-class model had a BIC score (9294.07) is not significantly different from that of 4, 5, and 6 class model, and lower than that of 1, 2, and 7 class model. The AIC drastically declines from 1 to 3 classes and then continued to decrease slightly thereafter. In addition to these empirical measures, for reasons of interpretability of the findings, and theory and prior findings in the literature, we chose the 3-class model as the final model.

### 4.3 Determining the optimal number of latent classes

Responses based on willingness to participate in the 13 carbon neutral activities were divided into 3 groups (see Table 2), each of which was given a name that attempted to reflect the behavioral probability of the visitors who constructed them regarding the question under investigation [79].

**Table 2:** Conditional response probability by class [response to participate].

item	class 1 (59.0 %)	class 2 (26.2 %)	class 3 (14.8 %)
Use of High-Efficiency Energy Products	0.965	0.774	0.628
Installation and Use of Renewable Energy Facilities	0.916	0.345	0.423
Efficient Operation of Heating and Cooling Systems	0.976	0.813	0.514
Enhancement of Home Insulation	0.939	0.489	0.441
Use of Public Transportation	0.852	0.499	0.460
Traveling by Bicycle, Walking	0.943	0.688	0.504
Use and Purchase of Eco-Friendly Vehicles	0.782	0.257	0.330
Avoiding Single-Use Products	0.979	0.956	0.124
Reducing Plastic Use	0.974	0.994	0.245
Reducing Food Waste	0.995	0.926	0.517
Using Second-hand Goods	0.876	0.564	0.469
Using Eco-Friendly and Low-Carbon Products	0.878	0.475	0.171
Using Locally Produced Food	0.911	0.628	0.350

#### 4.3.1 Group 1. active participation

This group shows a strong willingness to engage in all 13 carbon neutrality activities, more so than any other group. Their high level of participation suggests they have a deep understanding of carbon neutrality and how to reduce carbon emissions effectively, which translates into responsible, carbon-neutral behavior in their daily lives. This group represents 59.0 % of all respondents.

#### 4.3.2 Group 2. Limited Daily Life Participation

This group has a basic understanding of carbon neutrality and a somewhat positive attitude toward carbon-neutral practices. They are particularly willing to participate in activities that are relatively easy to incorporate into daily routines, such as 'efficient operation of heating and cooling systems,' 'avoiding single-use products,' 'reducing plastic use,' and 'minimizing food waste.' This group makes up 26.2 % of the respondents.

#### 4.3.3 Group 3. passive participation

This group shows a generally low willingness to participate in carbon neutrality activities compared to the other groups. They are less concerned about carbon neutrality and may require policy incentives or external monitoring to encourage even minimal participation. This group accounts for 14.8 % of the respondents.

**Fig. 4:**
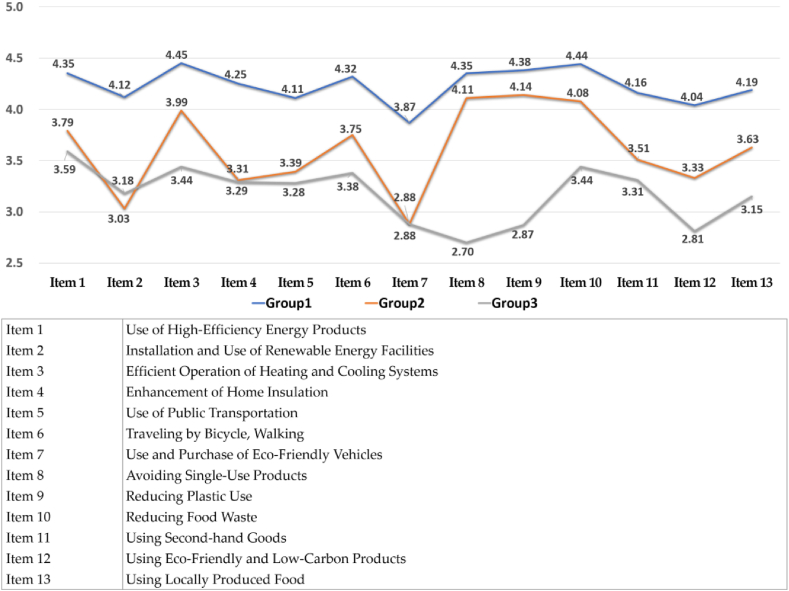
Comparison of averages by class.

Fig. 4 chart shows the willingness of three groups (Group 1, Group 2, and Group 3) to participate in various carbon neutrality activities. Group 1 generally shows the highest willingness across all activities, consistently scoring above the other groups. Group 2 shows moderate willingness, particularly for actions like using the efficient operation of high-efficiency energy heating and cooling systems, avoiding single-use products, and minimizing food waste. Group 3 displays the lowest willingness to engage in most activities, indicating a more passive approach to carbon-neutral behaviors. The activities include using renewable energy, enhancing home insulation, using public transportation, and reducing plastic use, among others.

### 4.4 Logistic regression analysis

To examine the differences between these three classes identified through latent class analysis, a specific group was compared with the rest, and logistic regression analysis was conducted. For this, the 'Active Participation Group' was recoded as 1, and other groups were recoded as 0 in the logistic regression analysis. The same recoding method was applied to analyze the 'Limited Daily Life Participation Group' and the 'Passive Participation Group' (see Table 3).

**Table 3:** Logistic regression analysis.

Independent Variables		Active Participation Group = 1, Other Groups = 0	Limited Daily Life Participation Group = 1, Other Groups = 0	Passive Participation Group = 1, Other Groups = 0
Independent Variables		Exp(B)	Exp(B)	Exp(B)
Awareness of the Climate Crisis	Degree of Impact of the Climate Crisis on Daily Life	1.656∗∗∗	0.548∗∗∗	1.091
	Evaluation of the Current Climate Crisis Situation	1.414∗∗	1.146	0.502∗∗∗
Practice of Responding to the Climate Crisis	Degree of Practice Among Peers	1.320∗∗∗	0.828∗∗∗	0.841∗∗
	Level of Awareness of Individual Actions in Response to the Climate Crisis	1.300∗	0.867	0.821
	Willingness to Bear Costs and Inconveniences for Achieving Carbon Neutrality	1.203∗	0.963	0.722∗
Control Variables	Gender	1.364	1.012	0.602∗
	Age	1.244∗∗	0.954	0.739∗∗
−2 Log Likelihood		943.019	839.092	602.709
Chi-Square		129.615∗∗∗	49.490∗∗∗	73.625∗∗∗
Nagelkerke R^2^		0.203	0.089	0.154

∗ < 0.05, ∗∗ < 0.01, ∗∗∗ < 0.001.

In comparing the groups based on their willingness to participate in carbon neutrality activities, differences between the 'Active Participation Group' and other groups were found to be positively influenced by 'the extent of the climate crisis impact on daily life' (B = 1.656, p < 0.001), 'evaluation of the current climate crisis situation' (B = 1.414, p < 0.01), 'degree of practice among peers' (B = 1.320, p < 0.001), 'awareness of individual-level actions in response to the climate crisis' (B = 1.300, p < 0.05), and 'willingness to bear costs and inconveniences for achieving carbon neutrality' (B = 1.203, p < 0.05). That is, the greater the impact of the climate crisis on daily life, the more serious the current climate crisis is perceived, the higher the level of practice among peers for carbon neutrality, the better the awareness of individual-level climate crisis actions, and the higher the willingness to bear costs and inconveniences, the more likely it is to be included in the 'Active Participation Group'. This is consistent with previous findings, Kwon [80] and Gebrehiwot and Anne [81] indicated that the more perceived severity, the higher the willingness to act in response to climate change. Chen [82] stated that a person's willingness to engage in energy-saving and carbon-reducing behaviors grows when they have a more positive attitude toward mitigating climate change and when their peers encourage them or join them in mitigation actions. Regarding individual-level, Yang et al. [83] stated that in societies with higher levels of individualism, environmental awareness is more likely to increase pro-environmental behavioral intentions and people will make greater efforts to protect the environment.

'Limited Daily Life Participation Group' and other groups, both 'the extent of the climate crisis impact on daily life' (B = 0.548, p < 0.001) and 'the degree of practice among peers' were found to have a negative impact (B = 0.828, p < 0.001). In other words, the lower the impact of the climate crisis on daily life and the lower the degree of carbon neutrality practices among peers, the higher the likelihood of belonging to the 'Limited Daily Life Participation Group'. As shown in Toole et al. [84], household adaptive behaviors that focus on everyday life increase when everyday households face climate change impacts. The impact of the degree of practices among peers on carbon neutrality-related behavior willingness has already been demonstrated in numerous studies, including Ma et al. [85]; Stevenson et al. [86].

In the case of the 'Passive Participation Group,' lower 'evaluation of the current climate crisis situation' (B = 0.502, p < 0.001), lower 'degree of practice among peers' (B = 0.841, p < 0.01), and lower 'willingness to bear costs and inconveniences' (B = 0.722, p < 0.05) were all associated with passive participation. This indicates that individuals who perceive the climate crisis as less severe, see less peer practice, and are less willing to bear costs are more likely to fall into the 'Passive Participation Group.' As shown in Tripathi and Mishra [87] and Patel et al. [88], many farmers believe that climate change is just a natural disaster and they passively adapt to it by making several changes in their agricultural activities. At the same time, the lower the willingness to bear costs and inconveniences, the higher the likelihood of being included in "passive participation". According to Bockarjova and Steg [89], the purchase of an electric vehicle by a user involved personal costs for the user, such as higher purchasing prices and costly behavioral adjustments related to driving and charging, and therefore governments around the world stimulate the introduction and adoption of electric vehicles, for example, by providing financial incentives.

Hypothesis 1 The degree impact of the climate crisis on daily life will affect the willingness to participate in activities for carbon neutrality.

This hypothesis is supported by the research findings. The logistic regression analysis revealed that the greater the impact of the climate crisis on daily life, the more likely individuals are to be classified within the 'Active Participation Group' (B = 1.656, p < 0.001). Conversely, a lower impact on daily life increases the likelihood of belonging to the 'Limited Daily Life Participation Group' (B = 0.548, p < 0.001).

Hypothesis 2 The evaluation of the current climate crisis situation will affect the willingness to participate in activities for carbon neutrality.

The results affirm this hypothesis. The evaluation of the current climate crisis situation was found to positively influence the likelihood of active participation (B = 1.414, p < 0.01). Additionally, a perception of the crisis as less severe increases the likelihood of passive participation (B = 0.502, p < 0.001).

Hypothesis 3 The degree of practice among peers will affect their willingness to participate in activities for carbon neutrality.

This hypothesis is validated by the findings. The study showed that the higher the degree of practice among peers, the more likely individuals are to engage in active participation (B = 1.320, p < 0.001). Conversely, lower peer practice is associated with a higher likelihood of belonging to both the 'Limited Daily Life Participation Group' (B = 0.828, p < 0.001) and the 'Passive Participation Group' (B = 0.841, p < 0.01).

Hypothesis 4 The level of awareness of individual actions in response to the climate crisis will affect the willingness to participate in activities for carbon neutrality.

The research findings support this hypothesis. Greater awareness of individual-level actions in response to the climate crisis increases the likelihood of active participation (B = 1.300, p < 0.05).

Hypothesis 5 The willingness to bear costs and inconveniences for achieving carbon neutrality will affect the willingness to participate in activities for carbon neutrality.

This hypothesis is also supported. The results indicate that a higher willingness to bear costs and inconveniences is associated with an increased likelihood of active participation (B = 1.203, p < 0.05). In contrast, a lower willingness to bear these costs and inconveniences is linked to a higher likelihood of passive participation (B = 0.722, p < 0.05).

All five hypotheses were confirmed, demonstrating significant relationships between factors such as the perceived impact of the climate crisis, peer behavior, individual awareness, and willingness to bear costs, and the likelihood of participating in carbon neutrality activities.

## 5 Conclusions

The climate crisis is a global issue that affects human survival and development, and addressing it requires the collective participation of all people. Low-carbon living habits also influence those around us, and the idea of sustainable, low-carbon environmental protection is gradually gaining traction [90]. In recent years, carbon neutrality efforts have garnered attention worldwide. Therefore, the focus of this paper is on how individual behaviors and social norms can change to foster positive actions toward achieving carbon neutrality in response to the climate crisis.

From the results, it's clear that both the 'Limited Daily Life Participation Group' and the 'Passive Participation Group' showed low levels of carbon-neutral practices among their peers. This indicates that responses to the climate crisis are shaped not only by individual awareness but also by the behaviors of those around them. To promote more active participation in climate crisis response, we need to explore strategies that engage not only individuals but also their social circles.

Additionally, the 'Passive Participation Group' tends to perceive the current climate crisis as less severe and shows a lower willingness to bear costs and inconveniences for carbon neutrality. Therefore, it is crucial to raise awareness about the serious impacts of the current climate changes and how failing to act now could lead to even greater costs and inconveniences in the future.

The study results show that awareness of the climate crisis and engagement in response practices have both direct and indirect effects on the willingness to participate in carbon-neutral activities. These findings offer several important insights as follows.

First, since personal awareness of the climate crisis influences willingness to engage in carbon-neutral activities, raising public awareness can inspire spontaneous environmental protection behaviors. As the public's understanding of the climate crisis deepens, so too will their ecological and environmental awareness. This makes it essential to promote environmental education, particularly focused on low-carbon living and carbon neutrality, to enhance public understanding of the climate crisis and increase support for carbon-neutral initiatives. Expanding awareness of low-carbon behaviors and environmental protection is a key way to boost public willingness to contribute to carbon neutrality [91]. Therefore, increasing public participation in carbon-neutral activities must start with improving individual awareness of the climate crisis. Effective policy measures should be developed to achieve this, including targeted communication and educational campaigns that engage the public and promote a clearer understanding of climate-related issues.

Second, an individual's environment and the actions of those around them significantly affect their willingness to engage in carbon-neutral activities. Personal participation is critical to achieving carbon neutrality, and individual actions are a vital force in tackling the climate crisis [92]. As we work to cultivate public awareness of carbon neutrality, we must encourage people to start with small actions in their daily lives, gradually integrating carbon-neutral practices into everyday habits. Governments can foster participation in carbon neutrality by promoting changes in consumption patterns and rationalizing energy use through economic incentives for energy-saving behaviors. This can motivate more energy-saving actions in response to the climate crisis. Conducting risk assessments of the local environment can also heighten public awareness of environmental protection and sustainable development, which can, in turn, drive greater participation in carbon-neutral activities [93]. Additionally, it is crucial to explore how individual behaviors can be shaped by community and social networks. The role of social norms, peer influence, and community-based initiatives in promoting pro-environmental behaviors and encouraging carbon neutrality efforts cannot be overlooked.

In conclusion, addressing the climate crisis requires not only raising awareness but also creating an environment in which individuals and communities are actively engaged in carbon-neutral practices. By implementing effective policies and leveraging the power of social networks, we can make significant strides in combating climate change and promoting sustainable development.

This study does have certain limitations. It primarily focused on how crisis awareness and the surrounding environment influence individuals' willingness to participate in carbon-neutral activities in South Korea. Other factors such as relevant policies, education level, income, age, and occupation were not examined [94,95]. Since the climate crisis impacts various aspects of people's daily lives globally, future research should expand the scope to include these factors and examine their influence on individuals' willingness to engage in carbon-neutral activities.

## CRediT authorship contribution statement

**Jae Eun Lee:** Supervision. **Seol A. Kwon:** Conceptualization. **Hyun Soo Park:** Software, Methodology. **Ke Zhang:** Writing – review & editing. **Wei Lu:** Writing – review & editing. **Lin Dong:** Writing – review & editing.

## Funding

This work was supported by the 10.13039/501100002701Ministry of Education of the Republic of Korea and the 10.13039/501100003725National Research Foundation of Korea (NRF-2023S1A5C2A02095270).

The authors do not have permission to share data.

## Declaration of competing interest

The authors declare the following financial interests/personal relationships which may be considered as potential competing interests: Jae Eun Lee reports financial support was provided by Ministry of Education of the Republic of Korea. If there are other authors, they declare that they have no known competing financial interests or personal relationships that could have appeared to influence the work reported in this paper.

## References

[1] United Nations Framework convention on climate change. https://unfccc.int/resource/docs/convkp/conveng.pdf.

[2] Kyoto Protocol to the United Nations Framework Convention on Climate Change. Available online: https://unfccc.int/resource/docs/convkp/kpeng.pdf (accessed on 1 December 2023). 1-21.

[3] The Paris Agreement. https://unfccc.int/sites/default/files/resource/parisagreement_publication.pdf.

[4] Provisional state of the global climate. https://wmo.int/publication-series/provisional-state-of-global-climate-2023.

[5] Di K., Chen W., Shi Q., Cai Q., Zhang B. (2024). Digital empowerment and win-win co-operation for green and low-carbon industrial development: analysis of regional differences based on GMM-ANN intelligence models. J. Clean. Prod..

[6] Zhang Y.H., Feng T.T. (2022). How does the design of personal carbon trading system affect willingness to participate under carbon neutrality goal?—evidence from a choice experiment. Environ. Sci. Pollut. Control Ser..

[7] Zhao S., Duan W., Zhao D., Song Q.I. (2022). Identifying the influence factors of residents' low-carbon behavior under the background of “Carbon Neutrality”: an empirical study of Qingdao city, China. Energy Rep..

[8] Zhang J., Lyu Y., Li Y., Geng Y. (2022). Digital economy: an innovation driving factor for low-carbon development. Environ. Impact Assess. Rev..

[9] Xue, H., Cai, M., Liu, B., Di, K., & Hu, J. Sustainable development through digital innovation: Unveiling the impact of big data comprehensive experimental zones on energy utilization efficiency. Sustain. Dev. 10.1002/sd.3112.

[10] Zhang Y., Fei X., Liu F., Chen J., You X., Huang S. (2022). Advances in forest management research in the context of carbon neutrality: a bibliometric analysis. Forests.

[11] Chen W., Li D., Cai Q., Di K., Liu C., Wang M. (2024). What influences the performance of carbon emissions in China?—research on the inter-provincial carbon emissions' conditional configuration impacts. PLoS One.

[12] Zhao D., Cai J., Xu Y., Liu Y., Yao M. (2023). Carbon sinks in urban public green spaces under carbon neutrality: a bibliometric analysis and systematic literature review. Urban For. Urban Green..

[13] Zhang S., Wang X., Xu J., Chen Q., Peng M. (2024). Green manufacturing for achieving carbon neutrality goal requires innovative technologies: a bibliometric analysis from 1991 to 2022. https://www.sciencedirect.com/science/article/pii/S1001074223003650.

[14] Fan J., Ding Z., Li K., Wang Q. (2023). Research on carbon neutrality from the past to the future: a bibliometric analysis. https://www.tandfonline.com/doi/abs/10.1080/14765284.2022.2116203.

[15] Xu X., Gou X., Zhang W., Zhao Y., Xu Z. (2023). A bibliometric analysis of carbon neutrality: research hotspots and future directions. Heliyon.

[16] Chen S., Liu J., Zhang Q., Teng F., McLellan B.C. (2022). A critical review on deployment planning and risk analysis of carbon capture, utilization, and storage (CCUS) toward carbon neutrality. Renew. Sustain. Energy Rev..

[17] Zhang Z., Hu G., Mu X., Kong L. (2022). From low carbon to carbon neutrality: a bibliometric analysis of the status, evolution and development trend. J. Environ. Manag..

[18] Guo Y.Y., Gou X.J., Xu Z.S., Skare M. (2022). Carbon pricing mechanism for the energy industry: a bibliometric study of optimal pricing policies. Acta Montan. Slovaca.

[19] Maestre-Andrés S., Drews S., Van den Bergh J. (2019). Perceived fairness and public acceptability of carbon pricing: a review of the literature. Clim. Pol..

[20] Tan X.C., Wang Y., Gu B.H., Kong L.S., Zeng A. (2022). Research on the national climate governance system toward carbon neutrality—a critical literature review. Fundamental Research.

[21] Tan X.C., Kong L.S., Gu B.H., Zeng A., Niu M.M. (2022). Research on the carbon neutrality governance under a polycentric approach. Adv. Clim. Change Res..

[22] Sun T., Di K., Shi Q., Hu J., Zhang X. (2024). Study on the development path of low-carbon retail clusters empowered by digital empowerment. J. Retailing Consum. Serv..

[23] Di K., Chen W., Shi Q., Cai Q., Liu S. (2024). Analysing the impact of coupled domestic demand dynamics of green and low-carbon consumption in the market based on SEM-ANN. J. Retailing Consum. Serv..

[24] Zhang L., Ling J., Lin M. (2023). Carbon neutrality: a comprehensive bibliometric analysis. Environ. Sci. Pollut. Control Ser..

[25] Di K., Chen W., Zhang X., Shi Q., Cai Q., Li D., Di Z. (2023). Regional unevenness and synergy of carbon emission reduction in China's green low-carbon circular economy. J. Clean. Prod..

[26] Liu S., Cai Q., Wang M., Di K. (2024). Urban public services and fertility intentions of internal migrants in China. PLoS One.

[27] Hao H., Qiao Q., Liu Z., Zhao F. (2017). Impact of recycling on energy consumption and greenhouse gas emissions from electric vehicle production: the China 2025 case. Resour. Conserv. Recycl..

[28] Buchanan K., Russo R., Anderson B. (2015). The question of energy reduction: the problem (s) with feedback. Energy Pol..

[29] Liu Y., Hong Z., Shi X. (2018). Antecedents of residents' repurchase intention of green residential building: case study of Sino-Singapore Tianjin ecocity. Energy Proc..

[30] Kurata Y., Ong A., Ang R., Angeles J., Bornilla B., Fabia J. (2023). Factors affecting flood disaster preparedness and mitigation in flood-prone areas in the Philippines: an integration of protection motivation theory and theory of planned behavior. Sustainability.

[31] Shafiei A., Maleksaeidi H. (2020). Pro-environmental behavior of university students: application of protection motivation theory. Global Ecology and Conservation.

[32] Whitmarsh L., Seyfang G., O'Neill S. (2011). Public engagement with carbon and climate change: to what extent is the public ‘carbon capable’?. Global Environ. Change.

[33] Wang Q., Song W., Peng X. (2022). The behavior-driven mechanism of consumer participation in “Carbon Neutrality”: based on the promotion of replacing coal with biomass briquette fuel. Int. J. Environ. Res. Publ. Health.

[34] Hügel S., Davies A.R. (2020). Public participation, engagement, and climate change adaptation: a review of the research literature. Wiley Interdisciplinary Reviews: Clim. Change.

[35] Lin Y., Anser M., Peng M., Irfan M. (2023). Assessment of renewable energy, financial growth and in accomplishing targets of China's cities carbon neutrality. Renew. Energy.

[36] Zhang J., Tong Z., Ji Z., Gong Y., Sun Y. (2022). Effects of climate change knowledge on adolescents' attitudes and willingness to participate in carbon neutrality education. Int. J. Environ. Res. Publ. Health.

[37] Mishra D., Akman I., Mishra A. (2014). Theory of reasoned action application for green information technology acceptance. Comput. Hum. Behav..

[38] Birkenberg A., Narjes M.E., Weinmann B., Birner R. (2021). The potential of carbon neutral labeling to engage coffee consumers in climate change mitigation. J. Clean. Prod..

[39] Wang F., Harindintwali J., Yuan Z., Wang M., Wang F., Li S., Chen J.M. (2021). Technologies and perspectives for achieving carbon neutrality. Innovation.

[40] Ajibade I., Armah F., Kuuire V., Luginaah I., McBean G. (2014). Self-reported experiences of climate change in Nigeria: the role of personal and socio-environmental factors. Climate.

[41] Spence A., Poortinga W., Pidgeon N., Lorenzoni I., Spence A., Poortinga W., Pidgeon N., Lorenzoni I. (2010). Public perceptions of energy choices: the influence of beliefs about climate change and the environment. Energy Environ..

[42] Wang T., Shen B., Springer C.H., Hou J. (2021). What prevents us from taking low-carbon actions? A comprehensive review of influencing factors affecting low-carbon behaviors. Energy Res. Social Sci..

[43] Tolppanen S., Kang J. (2021). The effect of values on carbon footprint and attitudes towards pro-environmental behavior. J. Clean. Prod..

[44] Wang Y., Guo C.H., Chen X.J., Jia L.Q., Guo X.N., Chen R.S., Wang H.D. (2021). Carbon peak and carbon neutrality in China: goals, implementation path and prospects. China Geology.

[45] Yuan X., Su C., Umar M., Shao X., LobonŢ O.R. (2022). The race to zero emissions: can renewable energy be the path to carbon neutrality?. J. Environ. Manag..

[46] Munasinghe M. (2011). Addressing sustainable development and climate change together using sustainomics. Wiley Interdisciplinary Reviews: Clim. Change.

[47] Kapsa I., Trempała W. (2020). Environmental awareness amongst youth in times of climate crisis. Athenaeum. Polskie Studia Politologiczne.

[48] Stoknes P.E. (2014). Rethinking climate communications and the “psychological climate paradox. Energy Res. Social Sci..

[49] Knight K.W. (2016). Public awareness and perception of climate change: a quantitative cross-national study. Environmental Sociology.

[50] Kwon S.A., Kim S., Lee J.E. (2019). Analyzing the determinants of individual action on climate change by specifying the roles of six values in South Korea. Sustainability.

[51] Jin Y., Liu B.F., Austin L.L. (2014). Examining the role of social media in effective crisis management: the effects of crisis origin, information form, and source on publics' crisis responses. Commun. Res..

[52] De La Peña L., Guo R., Cao X., Ni X., Zhang W. (2022). Accelerating the energy transition to achieve carbon neutrality. Resour. Conserv. Recycl..

[53] Batool N., Dada Z., Shah S.A. (2022). Developing tourist typology based on environmental concern: an application of the latent class analysis Mmodel. SN Social Sciences.

[54] Vermunt J.K., Magidson J. (2004). Latent class analysis. The Sage Encyclopedia of Social Sciences Research Methods.

[55] Porcu M., Giambona F. (2017). Introduction to latent class analysis with applications. J. Early Adolesc..

[56] Cui H., Zhang J., Zhu M., Sun W.R. (2024). Consideration of individual heterogeneity inactivity participation intention choice behavior of planned special events. J. Beijing Univ. Technol..

[57] McCubbin S., Smit B., Pearce T. (2015). Where does climate fit? Vulnerability to climate change in the context of multiple stressors in Funafuti, Tuvalu. Global Environ. Change.

[58] Epstein P.R., Ferber D. (2011).

[59] Bergquist M., Nilsson A., Schultz P. (2019). Experiencing a severe weather event increases concern about climate change. Front. Psychol..

[60] Reuter C., Hughes A.L., Kaufhold M.A. (2018). Social media in crisis management: an evaluation and analysis of crisis informatics research. Int. J. Hum. Comput. Interact..

[61] Ramyar R., Ackerman A., Johnston D.M. (2021). Adapting cities for climate change through urban green infrastructure planning. Cities.

[62] Li Y., Ye W., Wang M., Yan X. (2009). Climate change and drought: a risk assessment of crop-yield impacts. Clim. Res..

[63] Thomas K., Hardy R., Lazrus H., Mendez M., Orlove B., Rivera‐Collazo I., Winthrop R. (2019). Explaining differential vulnerability to climate change: a social science review. Wiley Interdisciplinary Reviews: Clim. Change.

[64] Huang S., Ma S., Pan Y., Li Y., Yuan Y., Tsai S.B. (2020). An empirical study on how climate and environmental issues awareness affects low carbon use behaviour. Ecological Chemistry and Engineering S.

[65] Wolf M., Serper M., Opsasnick L., O'Conor R., Curtis L., Benavente J., Bailey S.C. (2020). Awareness, attitudes, and actions related to COVID-19 among adults with chronic conditions at the onset of the US outbreak: a cross-sectional survey. Ann. Intern. Med..

[66] Dong H., Ma S., Jia N., Tian J. (2021). Understanding public transport satisfaction in post COVID-19 pandemic. Transport Pol..

[67] Shang W.L., Lv Z. (2023). Low carbon technology for carbon neutrality in sustainable cities: a survey. Sustain. Cities Soc..

[68] Paton D., Johnston D. (2001). Disasters and communities: vulnerability, resilience and preparedness. Disaster Prevention and Management. Int. J..

[69] Norris F., Stevens S., Pfefferbaum B., Wyche K., Pfefferbaum R.L. (2008). Community resilience as a metaphor, theory, set of capacities, and strategy for disaster readiness. Am. J. Community Psychol..

[70] Chen W., Li J. (2019). Who are the low-carbon activists? Analysis of the influence mechanism and group characteristics of low-carbon behavior in Tianjin, China. Sci. Total Environ..

[71] Levy B.S., Patz J.A. (2015). Climate change, human rights, and social justice. Annals of Global Health.

[72] Yang L., Li F., Zhang X. (2016). Chinese companies' awareness and perceptions of the Emissions Trading Scheme (ETS): evidence from a national survey in China. Energy Pol..

[73] Adaman F., Karalı N., Kumbaroğlu G., Or İ., Özkaynak B., Zenginobuz Ü. (2011). What determines urban households' willingness to pay for CO2 emission reductions in Turkey: a contingent valuation survey. Energy Pol..

[74] Larose C., Harel O., Kordas K., Dey D.K. (2016). Latent class analysis of incomplete data via an entropy-based criterion. Stat. Methodol..

[75] Weller B., Bowen N., Faubert S.J. (2020). Latent class analysis: a guide to best practice. J. Black Psychol..

[76] Nylund K., Asparouhov T., Muthén B.O. (2007). Deciding on the number of classes in latent class analysis and growth mixture modeling: a Monte Carlo simulation study. Struct. Equ. Model.: A Multidiscip. J..

[77] Larsen F., Pedersen M., Friis K., Glümer C., Lasgaard M. (2017). A latent class analysis of multimorbidity and the relationship to socio-demographic factors and health-related quality of life. A national population-based study of 162,283 Danish adults. PLoS One.

[78] Huh J., Riggs N., Spruijt‐Metz D., Chou C., Huang Z., Pentz M. (2011). Identifying patterns of eating and physical activity in children: a latent class analysis of obesity risk. Obesity.

[79] Batool N., Dada Z., Shah S.A. (2022). Developing tourist typology based on environmental concern: an application of the latent class analysis model. SN Social Sciences.

[80] Kwon S.A. (2022). Where does an individual's willingness to act on alleviating the climate crisis in Korea Arise from?. Sustainability.

[81] Gebrehiwot T., Anne van der Veen A. (2021). Farmers' drought experience, risk perceptions, and behavioural intentions for adaptation: evidence from Ethiopia. Clim. Dev..

[82] Chen M.F. (2016). Extending the theory of planned behavior model to explain people's energy savings and carbon reduction behavioral intentions to mitigate climate change in Taiwan–moral obligation matters. J. Clean. Prod..

[83] Yang M., Tang X., Cheung M., Zhang Y. (2021). An institutional perspective on consumers' environmental awareness and pro‐environmental behavioral intention: evidence from 39 countries. Bus. Strat. Environ..

[84] Toole S., Klocker N., Head L. (2016). Re-thinking climate change adaptation and capacities at the household scale. Climatic Change.

[85] Ma J., Zhou W., Guo S., Deng X., Song J., Xu D. (2022). Effects of conformity tendencies on farmers' willingness to take measures to respond to climate change: evidence from Sichuan Province, China. Int. J. Environ. Res. Publ. Health.

[86] Stevenson K., Peterson M., Bondell H.D. (2019). The influence of personal beliefs, friends, and family in building climate change concern among adolescents. Environ. Educ. Res..

[87] Tripathi A., Mishra A.K. (2017). Knowledge and passive adaptation to climate change: an example from Indian farmers. Climate Risk Management.

[88] Patel S., Mall R., Chaturvedi A., Singh R., Chand R. (2023). Passive adaptation to climate change among Indian farmers. Ecol. Indicat..

[89] Bockarjova M., Steg L. (2014). Can Protection Motivation Theory predict pro-environmental behavior? Explaining the adoption of electric vehicles in The Netherlands. Global Environ. Change.

[90] Dubois G., Sovacool B., Aall C., Nilsson M., Barbier C., Herrmann A., Sauerborn R. (2019). It starts at home? Climate policies targeting household consumption and behavioral decisions are key to low-carbon futures. Energy Res. Social Sci..

[91] Zhong S., Chen J. (2019). How environmental beliefs affect consumer willingness to pay for the greenness premium of low-carbon agricultural products in China: theoretical model and survey-based evidence. Sustainability.

[92] Seddon N., Smith A., Smith P., Key I., Chausson A., Girardin C., Turner B. (2021). Getting the message right on nature‐based solutions to climate change. Global Change Biol..

[93] Orsini F., Kahane R., Nono-Womdim R., Gianquinto G. (2013). Urban agriculture in the developing world: a review. Agron. Sustain. Dev..

[94] Cheng J., Li S., Wang Z., Cheng Z. (2022). The characteristic differences between ecological culture and low-carbon tourism cognition under the vision of carbon neutrality. Journal of Resources and Ecology.

[95] Zhang J., Zhang L., Qin Y., Wang X., Zheng Z. (2020). Influence of the built environment on urban residential low-carbon cognition in zhengzhou, China. J. Clean. Prod..

